# Kinesiological insights into exercise prescription within oncological prehabilitation: current knowledge and future perspectives

**DOI:** 10.3389/fspor.2025.1715484

**Published:** 2025-12-15

**Authors:** Giuditta Carretti, Andrea Tognaccini, Gabriele Baldini, Mirko Manetti, Mirca Marini

**Affiliations:** 1 Section of Anatomy and Histology, Department of Experimental and Clinical Medicine, University of Florence, Florence, Italy; 2 Multimodal Prehabilitation Center, Azienda Ospedaliero-Universitaria Careggi, Florence, Italy; 3 Section of Anesthesiology and Intensive Care, Department of Health Sciences, University of Florence, Florence, Italy

**Keywords:** applied kinesiology, adapted training, preoperative exercise, physiological reserve, cardiopulmonary exercise testing, cancer management, postoperative complications, multimodal prehabilitation

## Abstract

Surgery is currently considered one of the cornerstones of cancer care, though postoperative complications remain high and drastically impact on length of hospital stay, mortality, psychophysical recovery, quality of life, and healthcare costs. Preoperative functional capacity has been widely recognized as a reliable predictor of postoperative recovery. In the last 20 years, consistent evidence has shown that multimodal prehabilitation programs integrating tailored physical exercise can effectively improve postoperative outcomes and counteract surgical complications by increasing preoperative physiological reserve and cardiopulmonary efficiency. Nevertheless, field specific research shows substantial heterogeneity and lack of standardization of exercise prescription thus struggling to provide cancer/surgery type-based customization. On this basis, the present perspective article aims to provide a critical analysis of the available literature about exercise prescription within cancer prehabilitation to shed light on the optimal testing and training methodologies and describe the current and future potentialities of kinesiology in this clinical context. Our literature overview highlighted that high-quality multicenter trials applying validated kinesiological parameters/tools are needed to develop effective tailored training interventions, foster accessibility, and evaluate potential long-term benefits of cancer prehabilitation. Furthermore, to address the current knowledge gap and facilitate large-scale implementation of prehabilitation, global public investments should recognize the crucial role of kinesiologists. Indeed, a stable integration of such professional figures into multidisciplinary prehabilitation teams could help meet the multidimensional complex needs of oncological patients and safely promote long-term health benefits.

## 1 Introduction

Cancer represents one of the leading public health and socioeconomic challenges, ranking among the top three causes of death with an ever increasing incidence in the adult population worldwide (1–6).

Surgery remains the main effective cancer treatment further enhanced by novel neoadjuvant therapies and multidisciplinary approach to surgical oncological patients (7–9). Despite early diagnosis through screening progressively increased surgical eligibility of individuals thus reducing mortality (10–12), postoperative complications still occur in up to 40% of patients (13–15). Mostly unprepared to withstand the psychophysical and metabolic challenges due to operative procedures and perioperative treatments, one-third of patients present with modifiable preoperative risk factors contributing to postoperative morbidity and hindering recovery (16, 17). Research demonstrated that individuals with poor preoperative medical, psychophysical or nutritional status are more vulnerable to adverse outcomes (18–20). Complications are often linked to reduced functional ability, lower quality of life, prolonged hospital stays, increased mortality rates, and heightened healthcare costs, and may also delay adjuvant therapies, thus negatively impacting long-term survival (21–24). Cancer patients are more vulnerable to surgical stress and postoperative complications due to altered homeostasis, diminished aerobic capacity, and reduced muscle mass/strength (25, 26). Therefore, maintaining adequate preoperative physiological reserve is crucial for mitigating surgery-induced stressors and preventing unfavorable outcomes (27, 28). Neoadjuvant treatments may further deteriorate physical fitness, functional capacity, and nutritional health (8, 29), leading to treatment-related fatigue in up to 90% of patients (30, 31). Since oncological survivorship is increasing, a growing interest in ameliorating quality of life of cancer survivors is rapidly spreading.

In this context, prehabilitation was conceived and officially tested by the Prehabilitation Clinic of the McGill University Health Centre in the early 2000s (32). It specifically refers to a wide spectrum of interventions to optimize preoperative medical, psychophysical, and nutritional status of subjects scheduled for major surgery, thus facilitating recovery (27, 33–35). Prehabilitation exploits the preoperative time between cancer diagnosis and the planned surgical intervention (25, 36) as an opportunity to actively prepare patients through a unimodal or multimodal approach. Interventions frequently entail medical optimization, tailored exercise, nutritional counseling, cessation of dangerous life-style habits, and psychosocial support (32, 37). This complex approach requires both patient education/engagement and the solid expertise of different professionals collaborating in multidisciplinary teams (37, 38).

Regular physical activity is proven essential in oncology care to maintain or improve functional capacity during and following cancer treatment, counteract cancer-related psychophysical decline, and enhance quality of life (39, 40). Consequently, exercise protocols have been increasingly recommended and integrated into multidisciplinary cancer care pathways worldwide (41, 42). Physical exercise is defined as a structured and intentional form of training based on established parameters and eventually supplemented by respiratory exercises (43). Prehabilitation applies tailored physical training to effectively improve physiological reserve, cardiopulmonary efficiency, musculoskeletal functionality, and metabolic flexibility (44–46). Despite these recognized benefits, the major challenge to face in the oncological surgical population is the preoperative time constrained window (approximately 4 weeks from diagnosis to surgery). Therefore, tailored systematic training protocols are essential to meet the complex needs of these individuals (47–49). However, research lacks consistency in patient selection, adherence, testing/training methods, delivery strategies, and evaluated outcomes (50), hence struggling to provide standardized guidelines (9, 51, 52). Although existing evidence indicates that exercise regimens integrated into multimodal prehabilitation programs can positively influence postoperative outcomes (53, 54), further rigorous studies are required (21, 37, 55, 56).

Grounded on the American College of Sport Medicine (ACSM) Moving Through Cancer Program recommendations (57, 58), the present perspective seeks to contribute kinesiological insights into exercise prescription within oncological prehabilitation. By critically analyzing the available literature and leveraging the expertise of our research group in oncological prehabilitation (59, 60) and adapted exercise for cancer patients (31, 61, 62), we identified and discussed key strengths and limitations of the commonly employed testing/training methodologies, with the ultimate goal of highlighting the current and future potentialities of kinesiology in this promising field.

## 2 Assessment of preoperative fitness in prehabilitation

Oncological surgical complications are often linked to individual functional ability to cope with the increased metabolic demands and psychophysical needs induced by surgery (63, 64). It has been highlighted that poor cardiorespiratory fitness is associated with a higher risk of postoperative morbidity and mortality and delayed recovery (65–67), while a proper degree of preoperative physical resilience is correlated with better outcomes (68–70). Given the surgery-related trauma and stress response, preoperative functional reserve becomes a crucial parameter to satisfy these needs, improve postoperative outcomes, and predict complications (71–73). In such a perspective, personalized exercise can improve preoperative functional capacity thus fostering postoperative recovery (74, 75). The main international guidelines recommend tailoring exercise protocols to the individual health, psychophysical, and nutritional status. Consequently, a multimodal evaluation including validated tests should be performed to assess baseline functional capacity, especially when addressing vulnerable individuals (21, 76).

In particular, cardiopulmonary exercise testing (CPET) has been increasingly recognized as the gold standard to objectively assess cardiorespiratory fitness and exercise capacity in different healthcare settings, including multimodal prehabilitation (77, 78), thus allowing to efficiently and safely tailor preoperative exercise protocols (79–81). Furthermore, it is a reliable tool for perioperative risk stratification, enabling the optimal allocation of resources (21, 82). Exercise capacity is commonly assessed via oxygen consumption (VO₂) that reflects the efficiency in utilizing oxygen during physical activity (83). VO_2max_, the maximal oxygen uptake during incremental exercise, is a key marker of cardiopulmonary fitness and aerobic endurance (33, 82, 84) typically measured via CPET using a treadmill or cycle ergometer (85). When VO_2max_ is unattainable, due to health-related conditions, VO_2peak_ is reported as the highest VO₂ achieved (82, 83). Both have been linked to postoperative outcomes across surgical populations. Another relevant CPET parameter, the anaerobic threshold (VO_2AT_), indicates the shift to anaerobic metabolism. Such metabolic activation occurs in any type of tissue whenever the aerobic system is unable to supply ATP (adenosine triphosphate) at the required rate to meet the increasing energy demands (82, 85). Early VO_2AT_ detection helps identify individuals at risk of inadequate cardiopulmonary response during postoperative recovery (85, 86). In fact, an association between low anaerobic threshold values and post-surgery morbidity and mortality among cancer patients undergoing major surgery has been reported (67, 85, 87). In the context of multimodal prehabilitation, measuring the workload at which these values are achieved is helpful to prescribe tailored exercise programs, avoiding both under and overtraining (88). CPET is a validated tool across different clinical scenarios, but its application is frequently challenging in oncological patients due to cancer-related comorbidities or safety concerns (83, 89). Additionally, it requires specialized equipment and expertise which are not always accessible in all preoperative healthcare settings (83).

Alternatively, simpler and cost-effective tests like the Six-Minute Walk Test (6MWT) and Timed Up and Go (TUG) test are widely used to reliably evaluate preoperative functional capacity in individuals with reduced exercise tolerance and physiological reserve (90, 91). In the 6MWT the subject is asked to walk, at a sustained pace, along a 30-meter linear pathway for 6 min with the aim of achieving the longest distance (33, 71, 79). It moderately correlates with VO_2peak_ (92) and fairly with VO_2AT_ (91) and has demonstrated a reliable prognostic value in detecting susceptibility to postoperative complications (33, 48). The measurement of the perceived physical exertion during the 6MWT through the Borg Rating of Perceived Exertion scale, a validated purpose-designed qualitative tool, is frequently used for exercise prescription in prehabilitation (93–95). The TUG test assesses functional mobility, balance, and fall risk in a broad target of individuals. It measures the time needed to stand up from a chair, perform a 3-meter shuttle walk, and sit back down (71). Individuals showing a preoperative value more than 15 s are at higher risk of postoperative complications and long-term mortality after major surgery (96, 97). Walking tests are easily applicable in cancer patients due to their intrinsic features (i.e., intensity, executive pace, and eventual rest intervals completely and deliberately established by the subject) and the minimal required space, equipment, and expertise (90).

Other validated tests to measure functional capacity and strength have been used in trials investigating prehabilitation effectiveness, such as the Duke Activity Status Index (DASI), the Sit-to-Stand test, the handgrip strength test, and the Arm Curl test (32, 98, 99). DASI estimates psychophysical fitness through self-evaluation of the ability to perform several physical activities within an extended time frame, hence reflecting the overall functional capacity. It moderately correlates with VO_2peak_ and is useful to screen out surgical patients at high risk of major cardiovascular events and postoperative impairments (71, 100). The handgrip strength, Arm Curl, and Sit-to-Stand tests evaluate upper and lower limb musculoskeletal function and monitor the overall response to aerobic and resistance exercise in surgical patients involved in oncological prehabilitation (21, 72). In this context, preoperative frailty is frequently measured through validated scales investigating different domains and predicting adverse postoperative outcomes (44, 101). The Fried Frailty Phenotype Index is commonly used to measure physical frailty (102–104) allowing to objectively guide prehabilitation interventions (71, 105) and preoperative decision-making in high-risk vulnerable patients eligible for major surgery (106–108).

To clarify the practical role of exercise testing in oncological prehabilitation and encourage clinicians and stakeholders to effectively integrate applied kinesiology into preoperative care strategies, the main validated tools used to assess preoperative fitness are summarized in Table 1.

**Table 1:** Validated testing tools for assessing functional capacity and guide exercise prescription in cancer prehabilitation.

Testing tool	Features and aim	Main variables	Pros (References)	Cons (References)
Cardiopulmonary Exercise Testing (CPET)	Gold standard multidimensional assessment of cardiovascular, pulmonary, and musculoskeletal functionality/efficiency to evaluate submaximal and maximum/peak exercise physiological responses.	VO_2_ (individual ability to extract and use oxygen to produce energy at rest).	Non-invasive test.	Technically difficult to perform in unfit surgical patients due to cancer-related comorbidities and compromised exercise tolerance.
		VO_2max_ (maximum amount of oxygen utilized by the body during incremental exercise as key indicator of cardiopulmonary fitness and aerobic endurance).	Dynamic measurement of multidimensional parameters relevant for clinical decision making, identification of undiagnosed exercise intolerance (including differential diagnosis), and objective determination of individual preoperative functional capacity (21, 79, 80, 82, 85, 87)	Specific equipment, medical and technical expertise (83).
		VO_2peak_ (highest VO_2_ reached during CPET).		
		VO_2AT_ (anaerobic threshold).		
Six-Minute Walk Test (6MWT)	Validated submaximal exercise test widely applied to assess the cardiopulmonary function, aerobic capacity, and endurance.	6-min walking distance (maximum distance walked at a sustained pace along a 30-meter pathway in 6 minutes).	Well tolerated and safe.	Unable to identify the cause of exercise limitation.
	Moderately correlates with VO_2peak_.		Self-paced performance.	Results potentially influenced by several non-cardiopulmonary limitations (i.e., age, sex, weight, peripheral arterial disease, anemia, musculoskeletal impairments, and nutritional status).
			Good predictor of postoperative complications and mortality in oncological surgery.	Limited prognostic value in some specific health conditions.
			Useful to monitor prehabilitation response.	Learning and ceiling effect (91, 110–113).
			Minimal costs.	
			No specific equipment.	
			Useful for exercise prescription (30, 33, 71, 93, 94, 109).	
Timed Up and Go Test (TUG)	Validated assessing tool commonly administered in a wide range of population to evaluate/measure functional mobility, balance, and fall risk.	Total time needed to complete the task (rise from a chair, walk along a 3-meter linear pathway, turn around a set point, walk back and sit down).	Easy, quick, and low-cost administration.	Results potentially influenced by various factors (i.e., therapies side effects, blood pressure, footwear, eventual assistive devices, mental state/mood, balance impairments).
			Minimal equipment.	Does not provide insights about potential causes of gait and/or postural impairments (97, 114, 115).
			Good reliability in assessing balance and fall risk.	
			Good predictor of postoperative complications and long-term mortality in major cardiac and non-cardiac surgery (71, 96, 114).	
Sit-to-Stand Test (STS)	Validated tool to assess functional capacity, commonly used to evaluate lower limb strength and endurance, crucial parameters for maintaining daily life mobility and autonomy.	Total number of times the subject can stand up from and sit down in a chair within a specific time frame, or the time needed to complete a predefined set of repetitions.	Low-cost, easy and quick to administer.	Limited information about cardiovascular health and fitness.
			Minimal equipment required.	Outcome potentially influenced by various factors (i.e., balance and cognitive impairments, anthropometric characteristics, physical activity level).
			Widely applicable.	Scoring subjectivity (117, 118).
			Good predictor of postoperative complications and mortality in oncological surgical patients (21, 32, 72, 91, 116).	
Handgrip Strength Test (HGST)	Quantitative assessing tool widely used to measure the maximum voluntary isometric strength of the hand and of the forearm muscles.	Force exerted by the subject while squeezing a hand-held dynamometer as hard as possible (expressed in kilograms or pounds).	Simple, low cost, reliable and non-invasive tool.	Results potentially influenced by performing bias or errors, and/or by fatigue.
			Good predictor of muscle mass and/or sarcopenia risk, physical activity level, nutritional status, quality of life, functional autonomy, length of hospital stay, and mortality risk in a wide range of oncological surgeries (21, 32, 38, 71, 72).	Results representing muscle strength of the hand more than overall musculoskeletal strength/function.
				Accuracy potentially affected by hand size.
				Variability due to age, sex, and dominant hand.
				Lack of standardization due to different dynamometers and testing protocols.
Arm Curl Test	Validated test widely applied to evaluate upper body functional strength and endurance, particularly concerning muscles involved in lifting and curling motions (biceps, brachialis, and brachioradialis muscles).	Total number of successful repetitions within a preset time frame (generally 30 seconds).	Simple, quick, and low-cost.	Limited specificity due to a lack of muscle isolation.
			Minimal equipment.	Outcome potentially influenced by performing bias or errors, and/or fatigue, age, sex, and fitness level.
			Reliable assessment of the ability to perform everyday tasks involving the upper body (32, 72, 121).	Lack of accuracy for measuring pure muscle strength or endurance.
				Variable normative data (122–124).
Borg Rating Perceived Exertion (RPE) Scale	Visual/verbal numerical rating scale extensively used to measure and monitor the perceived effort during training or exercise testing (6MWT or CPET) without the need to rely on physiological parameters.	The 6 to 20 points Modified Borg Scale subjectively indicates by the subject the perceived exertion during the performed activity/task, taking into consideration physical stress and fatigue.	Simple, quick, and low-cost.	Results potentially influenced by contingent psychophysical factors (motivation, fatigue, mood, depression).
			Minimal equipment.	Potential misinterpretation of the perceived effort.
			Easily accessible.	Education and guidance needed to well understand the Numeric/visual scale and correctly refer these values to different perceived efforts (125, 126).
			Good correlation with heart rate, oxygen consumption, and other physiological parameters during exertion.	
			Useful for educating patients to adequately and self-exercise according to the prescribed intensity (95).	
Duke Activity Status Index (DASI)	Self-administered questionnaire widely applied in clinical practice to assess functional capacity.	Total score obtained by responding to 12 items focused on various activities of daily living, performed in the last week.	Self-administered, quick and low-cost.	Subjective.
	Moderately correlates with VO_2peak_.		Useful for decision making within a wide range of clinical settings/populations.	Limited scope concerning the full spectrum of daily life activities.
			Good predictor of mortality, cardiovascular morbidity and disability, in several health conditions.	Outcomes potentially influenced by cognitive impairments.
			Reliable insights about limitations in performing specific activities of daily living (32, 71, 98).	Limited sensitivity for detecting minor changes in functional capacity, especially in stabilized outcomes (98).
Fried Frailty Phenotype Index (FFPI)	Validated assessing tool, based on 5 main features, widely used in various clinical settings to identify and classify physical frailty.	Total score obtained by recording each of the five prevised criteria as present or absent (binary coding).	Easily and quickly administered.	Based on specific parameters not routinely assessed in many clinical settings (i.e., preoperative clinics).
			Extensive validation in a wide range of medical and surgical populations.	Limited scope in capturing psychosocial aspects of frailty.
			Good predictor of mortality, morbidity, delirium, postoperative cognitive impairments, falls, hospitalizations, disability, and other adverse clinical outcomes.	Less suitable in case of functional limitations/disabilities (104, 127).
			Objective measurements (54, 104, 106, 107).	

## 3 Kinesiological approach and main applied training methodologies

The Enhanced Recovery After Surgery (ERAS®) Society advocates prehabilitation to improve preoperative fitness of oncological surgical patients and recommends structured exercise to optimize psychophysical functionality and foster postoperative recovery (53, 128).

It has been demonstrated that preoperative exercise interventions provide significant psychophysical benefits in cancer surgical patients by improving cardiorespiratory fitness, quality of life, daily life functionality, and promoting long-term health behavioral changes (55). Despite these demonstrated benefits, literature struggles to provide a general consensus or standardizable practical guidance (32, 89, 129). The global initiative “Exercise is Medicine” promoted by ACSM aims to integrate physical activity/exercise into standard clinical care to prevent and manage several diseases (76, 130).

As generally required for any medication, even exercise should be individually prescribed and monitored (33). Therefore, the ACSM guidelines recommend to strictly follow the Frequency, Intensity, Timing, Type plus Volume, and Progression (FITT-VP) principles when prescribing exercise, particularly for vulnerable subjects (130, 131). Within oncological prehabilitation, exercise work-rate should be individually set and monitored, based on objective measures of functional capacity (33, 74, 84) thus yielding the greatest benefits in the available time constrained window (33, 47).

Supervised exercise programs, face-to-face or small group settings, combining aerobic and resistance exercises, seem to be more effective than self-leaded ones at facilitating adherence to the protocols and postoperative functional recovery (132–134), especially in frail and high-risk oncological patients (21, 135). However, several studies reported that logistical and cost-related factors might hinder accessibility when conducted in clinical settings (136, 137). Kinesiologists own specific knowledge and competences that, if structurally integrated into a multidisciplinary prehabilitation team, might significantly facilitate the widespread implementation of preoperative exercise, regardless of the setting (52, 138). Despite possible differences related to country, regulatory body, and subjective specialization, the core competences generally acquired and trained by kinesiologists are multidisciplinary oriented. In detail, purposely designed University programs previse the acquisition of foundational knowledge in the biomedical field practically applied to exercise physiology and movement science. Psychology, pedagogy, and research methodology must be also mastered to effectively foster and support behavior changes and long-term adherence to exercise programs by variegated target of individuals. Kinesiologists are graduated multicompetent professionals highly specialized in designing, customizing, leading, and monitoring evidence-based training/testing protocols in a wide range of contexts. Therefore, they play a pivotal role in exercise prescription, administration, and evaluation tailored to multimodal prehabilitation programs.

Exercise programs tailored to surgical cancer patients can be classified into two main categories: endurance (moderate continuous and high intensity interval training) and resistance training. Additionally, integration of breathing exercises is a recognized effective strategy to mitigate potentially postoperative respiratory complications after major surgery, particularly in high-risk patients (89, 129, 139).

Table 2 summarizes the main physical exercise components of multimodal prehabilitation by categorizing them as follows: (i) aerobic training, namely Moderate Intensity Continuous Training (MICT) and High Intensity Interval Training (HIIT), (ii) resistance training, and (iii) breathing exercises. The table was conceived based on exercise science principles widely accepted and applied by kinesiologists in the clinical context following the main international guidelines to design and tailor training programs (130). Hopefully, it could help promote a scientific approach to exercise prescription in this sensitive clinical context.

**Table 2:** Features and criticalities of different training methodologies based on kinesiology cornerstones and applied in cancer prehabilitation.

Evidence-based training methodology	Training features and main objectives	Main investigated cancer type (References)	Overall demonstrated benefits in the context of oncological prehabilitation (References)	Limitations in the context of oncological prehabilitation (References)
Moderate Intensity Continuous Training (MICT)	Primary aim: sustained endurance exercise at moderate intensity (60-80% of maximal exercise capacity) to improve cardiorespiratory efficiency and overall fitness.	Breast (140–142).	Effective and well-tolerated training methodology to improve cardiorespiratory fitness, functional capacity, psychophysical well-being, and postoperative recovery.	Scarce use of CPET to establish and personalize exercise prescription.
	Secondary aims: chronic disease risk and fatigue reduction, weight management, mood and quality of sleep enhancement.	Lung (37, 111, 143, 144).	No major adverse events during supervised exercise sessions (33, 47, 48, 134, 138, 157).	Heterogeneity of the studied population.
	Duration and frequency of exercise sessions not standardized;	Esophagogastric (30, 145, 146).		Lack of monitoring during prehabilitation and long-term adherence.
	2-3 weekly sessions of 20-60 minutes, integrated into a multimodal exercise regimen, are commonly prescribed.	Pancreatic (147–151).		Risk of duration-related boredom (25, 33, 129, 136, 158).
		Liver (151, 152).		
		Colorectal (33, 80, 90, 153–156).		
High-Intensity Interval Training (HIIT)	Primary aim: aerobic training alternating short bursts of high intensity (85-95% maximal exercise capacity) and low intensity (40-60% maximal exercise capacity) workout to improve cardiovascular efficiency, aerobic/anaerobic capacity, muscle mass, insulin sensitivity, and metabolism.	Lung (89, 159, 160).	Effective and time-efficient training methodology to improve aerobic and anaerobic threshold, cardiac systolic and diastolic function, muscle strength, and attenuate fatigue.	Lack of standardization.
	Secondary aims: fat loss fastening, stress and anxiety reduction, optimize workout time.	Pancreatic (161).	Long-term maintenance of psychophysical training benefits.	Heterogeneity of the studied population.
	Duration and frequency of exercise sessions not standardized; 2-3 weekly sessions of 20-40 minutes (short warm-up, 4 HIIT intervals of 2-3 minutes each, and a few minutes cooldown), integrated into a multimodal exercise regimen, are commonly prescribed.	Colorectal (18, 33, 90).	No major adverse events during supervised exercise (47, 90, 138, 159, 165–168).	Potential difficulties in reaching and managing the established work rate prescribed in case of non-supervised training.
		Liver (151, 162).		Potential risk of not tolerating high-intensity exercise training in specific cancer types and patients (malnourished, sarcopenic, frail, anemic) or in patients receiving neoadjuvant therapies.
		Urologic (163, 164).		Sometimes perceived psychophysically challenging (25, 52, 89, 134, 136, 138).
Resistance Training	Primary aim: muscular work against external resistance to improve muscle strength/mass, bone density, anaerobic endurance, and metabolism.	Breast (140–142, 169).	Effective and safe training methodology to counteract cancer-related fatigue and sarcopenia, promote postoperative functional recovery, psychophysical well-being, and daily life autonomy.	Lack of standardization.
	Secondary aims: prevention of joint injury and sarcopenia, improvement of free fat mass index, improve tolerance to cancer-related therapies, stress and anxiety reduction.	Lung (37, 111, 143).	No major adverse events or injury during supervised exercise (33, 48, 134, 172, 173).	Heterogeneity of the studied population.
	Duration and frequency of exercise sessions not standardized; 2-3 weekly sessions at 40-50% of the 1RM (1-4 sets of 8-12 repetitions each involving major muscle groups), integrated into a multimodal exercise regimen, are commonly prescribed.	Esophagogastric (30, 145, 146).		Equipment availability/costs.
		Pancreatic (147–151).		Proper executive techniques are required.
		Colorectal (18, 33, 80, 153, 154).		Potential difficulties in following the preset workload in case of non-supervised training, due to fatigue-related issues (25, 136, 172).
		Urologic (170, 171).		
Breathing Exercises	Primary aim: techniques to improve respiratory function (inspiratory muscle training based on measured Maximal Inspiratory Pressure-MIP, improvement of lung capacity, and respiratory dynamic obstruction).	Lung (37, 143, 174).	Effective integrative training methodology to reduce postoperative respiratory complications and promote overall recovery and exercise tolerance.	Heterogeneity of the studied population.
	Secondary aims: improvement of overall body awareness, sleep quality, and management of stress induced by surgery (i.e., mindfulness).	Esophagogastric (175).	Great accessibility (except for Inspiratory Muscle Training for which specific devices are needed), cost-effective.	Training sessions to ensure adequate adherence, and tailored supervision if needed.
	Prescription not standardized; variable daily sessions within a multimodal exercise regimen are commonly prescribed.	Colorectal (155).	High-adherence rate.	Need for specific devices (i.e., Inspiratory Muscle Training) (89, 178).
			No adverse events/injury risk among oncological patients (89, 174, 176–178).	

### 3.1 Aerobic training

Aerobic training, comprising MICT and HIIT, is the most frequently used exercise type in prehabilitation programs since it can effectively and safely improve preoperative oxygen consumption (33, 48, 134).

MICT involves performing exercise continuously at a submaximal work rate while HIIT alternates high- and low-intensity bouts (90, 167, 179). Both are valid strategies to enhance preoperative functional capacity in cancer patients (160, 180), but HIIT better improves VO_2peak_ and cardiac function within the limited preoperative window (55, 159, 165, 168) and is associated with long-term postoperative benefits (47, 181). Proper warm-up remains essential to mitigate injury and cardiovascular risk (89, 134). A combination of moderate-to-high-intensity aerobic and anaerobic exercise performed in 30–60 min sessions, scheduled 3–4 times per week, for a median duration of 4 weeks, represents the most effective exercise prescription to improve functional capacity (133, 182). Though the type, dose, and schedule of prehabilitation exercise interventions widely varied across the available literature, trials utilizing CPET-derived variables to set intensity thresholds frequently reported better postoperative outcomes (33, 158).

### 3.2 Resistance training

Resistance training increases muscle strength and mass by stimulating muscular work against a weight/force administered through free weights, isotonic/isokinetic machines, resistance bands, or body weight (33, 134). In cancer patients, muscle loss due to the disease, neoadjuvant therapy, and surgical stress makes resistance training a key component of prehabilitation (48). However, guidelines on optimal training intensity, typically measured by the one-repetition maximum (1 RM), are limited (134, 172, 183). Currently, one to 4 sets of 8–12 repetitions each, performed at an intensity of 40%–50% of 1 RM, are recommendable to improve muscular strength and mass in vulnerable subjects (172, 173). Specifically referring to oncological patients, circuit training targeting major muscle groups enables personalized load adjustments while preserving the social and emotional benefits of group training (61, 178).

### 3.3 Breathing exercises

Respiratory training is a valuable adjunct to counteract surgery-related pulmonary complications by improving lung function and oxygenation, hence promoting postoperative functional recovery (89, 184) and reducing hospital stay (174, 176).

Conscious breathing techniques are also important for managing distress and anxiety, thus helping cancer patients to better overcome surgery-related stressors, and enhance psychophysical resilience (31, 62). Daily inspiratory/expiratory muscle training and volumetric variations can positively affect the preoperative forced expiratory volume in 1 s (FEV1), hence counteracting morbidity and mortality (177, 185). However, only few studies provided a detailed exercise regimen to improve respiratory function, including 1–3 daily sets of 10–15 repetitions of abdominal or pursed-lip breathing (178). Despite the highlighted overall benefits, breathing exercises are often administered only to specific cancer populations (74, 89, 140, 174).

## 4 Synthesis of evidence-based recommendations and applications

According to scientific evidence and our on-field expertise, structured exercise testing and training interventions tailored to cancer prehabilitation can positively affect postoperative outcomes in surgical patients (60, 80, 157). Even though literature shows that exercise represents the most effective component of oncological prehabilitation, its integration within multimodal programs including nutritional and psychological counseling appears to be the best choice in this field (75, 182). In fact, such multidisciplinary patient management can safely and effectively meet the multidimensional complex needs of this vulnerable population. Nevertheless, evidence-based customization for different cancer types, as well as protocol standardization, both for testing and training, are currently not available. These criticalities are mostly due to the wide population/treatment heterogeneity (50, 55), and a general lack of acknowledgement of the kinesiologist by the healthcare system. Currently, gastrointestinal cancer affected individuals scheduled for major surgery represent the most investigated population in the context of oncological prehabilitation (71, 80, 182), but application and research addressing other cancer types are progressively emerging. Concerning workout methodology, it has been highlighted that CPET derived outcomes allow to safely and objectively customize training interventions to the individual cardiorespiratory fitness and exercise capacity (33, 79, 80). Furthermore, such a gold standard tool, by investigating the main ventilatory thresholds, can reliably drive HIIT sessions design, administration, and monitoring. Despite literature evidenced that exercise protocols tailored to cancer patients should integrate endurance, interval, and resistance training, HIIT constitutes the most effective methodological approach to optimize functional capacity within the short-time preoperative window (156, 160, 165, 166). Since HIIT does not always fit the reduced exercise tolerance of oncological patients and it is physically challenging, supervised sessions seem to be the best delivery method (136, 137). On this basis, it is the authors' opinion that the previously detailed multidisciplinary competences of kinesiologists become crucial within physical exercise prescription and administration in this vulnerable target population.

## 5 Discussion and future directions

According to the available literature, multimodal prehabilitation programs entailing structured exercise (44, 75, 180, 182) can positively affect preoperative physiological reserve and postoperative outcomes in surgical cancer patients (33, 80, 157). Nevertheless, widespread adoption of evidence-based and standardized exercise protocols remains limited, probably due to the recent introduction of oncological prehabilitation science and the necessity of customizing interventions to such heterogenous population (50, 55). In particular, multicenter randomized trials applying FITT-VP parameters and monitoring exercise compliance are essential to better clarify the efficacy of cancer prehabilitation (52, 136, 186). Regarding future research directions, studies should explicitly report the professional figures involved in exercise prescription and administration within oncological prehabilitation. Such methodological clarity might help compare the efficacy of different competences and optimize role and resources assignment in this complex and multidisciplinary context. Promoting evidence-based exercise prescription, standardizing exercise programs, and implementing them into clinical practice may help advance prehabilitation and expand its application across a wide range of clinical settings (105, 187, 188).

It is the authors' opinion that the significant competences in delivering exercise science held by kinesiologists might overcome the main highlighted critical issues, and their routine integration into multiprofessionals prehabilitation teams might potentially facilitate a large-scale implementation of exercise programs, thus further improving prehabilitation efficacy. The ACSM exercise guidelines for oncology underlined that protocol adherence is essential for achieving long-term behavioral changes and health benefits (57, 58). In the prehabilitation context, adherence is often jeopardized by a lack of competent supervision, both during testing and training (130). Considering the sub-optimal adherence to prehabilitation programs (189), kinesiologists' support could help patients to safely overcome their reduced exercise tolerance and reach the required work rates within the time constrained preoperative window (132, 134). However, prehabilitation programs routinely employing kinesiologists are scarcely available.

Several studies highlighted the effectiveness of HIIT in enhancing preoperative physiological reserve and counteracting postoperative complications (156, 160, 166). Nevertheless, cancer-related issues might hinder this type of exercise (136, 137), thus further supporting the engagement of kinesiologists to safely deliver, supervise, and monitor exercise in this vulnerable population (33, 89, 129, 172). Moreover, the specific on-field trained competences of kinesiologists may be crucial for proactively engaging surgical patients in exercise programs, promoting long-term behavioral changes, and fostering independent exercise management.

Evidence-based kinesiology is a relatively recent discipline, and legislation regulating implementation of this science into clinical practice widely varies worldwide. Based on the double expertise of our group in the academic and clinical field, specific guidelines concerning University programs to train kinesiologists might help standardize their educational background worldwide. Achieving a greater uniformity in the field-specific core competences might facilitate the classification and acknowledgement of such emerging professional category. Furthermore, we support and recommend integrating prehabilitation specific training within the aforementioned University programs to ease the practical application of the kinesiological competences within this clinical frame. Presently, kinesiologists are not classified as healthcare professionals, hence hampering their inclusion within clinical frames. Consequently, preoperative exercise is often led and supervised by healthcare professionals, thus inducing patients to perceive it as a contingent medical treatment rather than a healthy life-habit to be acquired. In future perspective, regulatory agreements between healthcare authorities, facilities, and university programs, recognizing the kinesiologists' expertise and utility, might facilitate their integration into multidisciplinary teams, as in the context of oncological prehabilitation. This kind of institutional initiative could help spread research interest and possibly overcome the shortage of several health-related professional figures required to implement exercise medicine. Finally, the development of hospital and community-based structured exercise programs, supervised by specialized kinesiologists, might foster exercise adherence and benefits during the whole continuum of cancer care (190), thus helping these vulnerable individuals to regain baseline function and quality of life while positively impacting on healthcare and societal costs (191–193).

## Data Availability

The original contributions presented in the study are included in the article/Supplementary Material, further inquiries can be directed to the corresponding author.
